# First case report of onychomycosis caused by *Exophiala alcalophila*

**DOI:** 10.1016/j.mmcr.2026.100820

**Published:** 2026-09-03

**Authors:** Lili Rueda-Jaime, Leticia Sopó-Prada, Carolina Firacative

**Affiliations:** a Clinical and Infectious Dermatology Research Group, Universidad El Bosque, Bogota, 110121, Colombia; b Laboratorio de Micología Médica, Bogota, 110121, Colombia; c Studies in Translational Microbiology and Emerging Diseases (MICROS) Research Group, School of Medicine and Health Sciences, Universidad del Rosario, Bogota, 111221, Colombia

**Keywords:** *Exophiala*, Onychomycosis, Antifungal resistance, Black yeast-like fungi, Colombia

## Abstract

A 46-year-old woman from Colombia, without any medical comorbidities, presented with marked yellowish discoloration of both big toenails, suggestive of onychomycosis, and malalignment of the nail plate. Culture of nail scrapings grew dark-pigmented, mucoid, yeast-like colonies identified as *Exophiala alcalophila* by ITS sequencing. Broth microdilution identified the isolate as resistant to fluconazole and the echinocandins. The patient refused antifungal treatment. This case highlights the importance of black yeast-like fungi as etiological agents of onychomycosis.

## 1 Introduction

*Exophiala* is a genus of dematiaceous fungi ubiquitously present in the environment, currently comprising more than 60 species, with *Exophiala dermatitidis*, *Exophiala jeanselmei*, and *Exophiala spinifera* being the most prevalent [1,2]. Within the genus, 26 species have been documented as human pathogens, causing infections ranging from skin and subcutaneous tissue infections to life-threatening systemic disease, such as endocarditis and infections of the lungs, blood, and the central nervous system, especially in immunocompromised patients [1,3]. In addition, in recent decades, *Exophiala* species have been increasingly recognized as responsible for both superficial and disseminated infections in healthy individuals, including mycetoma and other clinical forms of phaeohyphomycosis [4].

In dermatological practice, dermatophytes, and, to a lesser extent, yeast and a small number of filamentous fungi, are primarily reported to cause cutaneous and superficial fungal infections of the skin and nails [5,6]. Members of black yeast-like fungi, such as *Exophiala*, have also been reported to cause subcutaneous and superficial skin infections. In fact, the top three affected sites in Exophiala infections are the subcutaneous tissue, lungs, and skin [4]. However, in onychomycosis, which is a chronic fungal nail infection with high prevalence worldwide, *Exophiala* species are rather rare [6]. To date, four cases of onychomycosis caused by *E. dermatitidis*, three each by *E. jeanselmei* and *Exophiala oligosperma*, and one each by *Exophiala bergeri* and *Exophiala hongkongensis* have been reported in the literature [3,[7], [8], [9], [10], [11], [12], [13], [14]] (Table 1). To our knowledge, however, this is the first time that *Exophiala alcalophila* is reported as a causal agent of onychomycosis in the world, notably in a healthy patient. This species, very uncommon in the genus *Exophiala*, has a restricted geographical distribution, isolated scarcely from soil, households, and human skin, but never causing disease in human hosts [1,2]. This case report is therefore very interesting due to the rarity of such infections, calling upon dermatologists to pay attention to black yeast-like fungi, to establish their role in superficial infections, such as onychomycosis, even in the immunocompetent.

**Table 1:** Cases of onychomycosis caused by *Exophiala* species, including those reported in the literature and the case reported herein.

Country/Year	Species	Age/Sex	Risk factor	Treatment/Duration	Response	Ref.
Japan, 1992	*E. dermatitidis*	51/F	Diabetes mellitus	1% topical bifonazole solution, 4 months	Recovered	[7]
Japan, 1999	*E. dermatitidis*	61/F	None	Topical terbinafine hydrochloride, 4 months	Recovered	[8]
France, 2002	*E. jeanselmei*	60/M	Renal transplant	Terbinafine, 6 months. Itraconazole, 6 months	Recovered	[9]
Morocco, 2009	*E. jeanselmei*	39/F	Immunocompromised	Itraconazole, 3 months^a^	Recovered	[10]
South Korea, 2011	*E. dermatitidis*	42/M	None	Itraconazole, 6 months	Recovered	[11]
India, 2012	*E. jeanselmei*	50/M	None	Itraconazole, ND	Recovered	[12]
China, 2013	*E. bergeri*	37/M	None	ND	ND	[3]
	*E. oligosperma*	23/M	None	ND	ND	
	*E. oligosperma*	51/F	None	ND	ND	
	*E. hongkongensis*	68/F	Hypertension	ND	ND	
Taiwan, 2016	*E. oligosperma*	54/F	None	Sulconazole nitrate, ND	ND	[13]
Brazil, 2023	*E. dermatitidis*^b^	50/ND	None	Itraconazole, 4 months	Bad response	[14]
Colombia, 2025	*E. alcalophila*	46/F	None	None	NA	This study

a The patient was treated with 150 mg fluconazole/week for 1 year without any improvement.

b Coinfection with *Candida parapsilosis*. *Exophiala* (*E*.)*;* Female (F); Male (M) No data (ND); not applicable (NA).

## 2 Case presentation

**Fig. 1:**
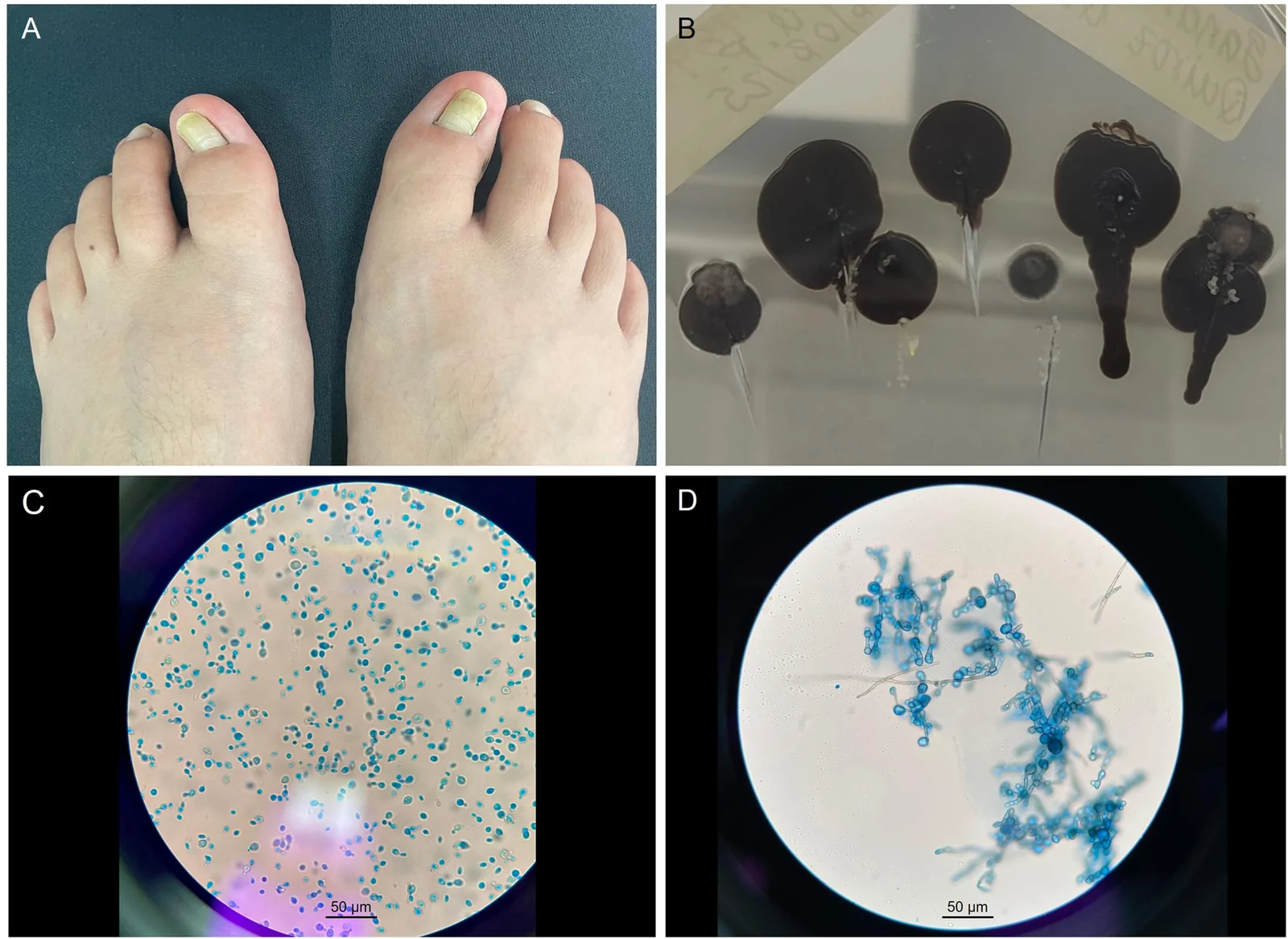
Clinical and microbiological characteristics of onychomycosis caused by *Exophiala alcalophila*. Yellowish discoloration of the left and right big toenails and malalignment of the nail plates (A). Dark-pigmented, mucoid, yeast-like colonies resulting from culture of nail scrapings (B), which, after staining with lactophenol cotton blue, were observed microscopically (400X) as abundant budding yeasts (C) and sparsely septate torulose hyphae (D).

A 46-year-old female patient with no significant medical comorbidities presented with a 20-year history of changes in toenail coloration (day 0). She reported undergoing multiple procedures on the big toenails several years ago due to recurrent onychocryptosis. Following these procedures, the patient noticed persistent changes in nail color and received itraconazole pulse therapy for three months, which did not improve the condition. Physical examination of the toenails revealed marked yellowish discoloration of both big toenails, suggestive of onychomycosis, and malalignment of the nail plate (Fig. 1A). The other nails were normal, as was the skin of the soles and interdigital webs. Toenail sampling was performed on the two involved nails, taking eight to ten nail clippings along with subungual debris to improve the accuracy of diagnosis. Direct examination of nail scrapings with KOH and Parker ink was negative. However, on day 30, cultures of all nail samples from both toenails, on Sabouraud's dextrose agar containing chloramphenicol and gentamicin with cycloheximide, incubated at 25**°**C, yielded dark-pigmented, mucoid, yeast-like colonies (Fig. 1B). The culture, observed microscopically with lactophenol cotton blue, showed abundant budding yeast-like cells and sparsely septate torulose hyphae (Fig. 1C and D). No other fungi, including dermatophytes, yeasts, and environmental molds, grew on the cultures.

While matrix-assisted laser desorption/ionization time-of-flight (MALDI-TOF) mass spectrometry failed to identify the dematiaceous fungus, species identification was achieved by amplification and sequencing of a section of the nuclear fungal ribosomal DNA, performed according to the International Society for Human and Animal Mycology (ISHAM) barcoding scheme with the primers SR6R (AAGTARAAGTCGTAACAAGG) and LR1 (GGTTGGTTTCTTTTCCT) [15]. These primers amplify the very end of the small subunit (SSU) of the ribosomal RNA gene, the full internal transcribed spacer 1 (ITS1), the 5.8S ribosomal RNA gene, the ITS2, and the 5’ end of the large subunit (LSU) of the ribosomal RNA gene. PCR products were commercially purified and sequenced, both forward and reverse strands, by GenCore, Universidad de los Andes, Bogota, Colombia. Sequences were edited, and contigs were assembled using the program Sequencher 5.4.6 (Gene Codes Corporation, USA). The sequence of the isolate, comprising 658 base pairs, identified as *Exophiala alcalophila*, was deposited in GenBank under the accession number PX935142.

Antifungal susceptibility testing of the isolate against nine antifungals was performed using the colorimetric broth microdilution test, Sensititre® YeastOne® plates (Thermo Scientific, Waltham, MA, USA), following the recommendations of the M27M44S guideline of the CLSI [16]. The isolate of *E. alcalophila* was confirmed to be resistant to anidulafungin, micafungin, and caspofungin, considering that this is a basidiomycetous species and that the minimal inhibitory concentration (MIC) for all three echinocandins was >8 μg/mL. In addition, the isolate was resistant to fluconazole (MIC >256 μg/mL) and susceptible to amphotericin B (MIC = 0.125 μg/mL), flucytosine (MIC <0.0625 μg/mL), and the triazoles tested, posaconazole (MIC = 0.03125 μg/mL), voriconazole (MIC = 0.25 μg/mL), and itraconazole (MIC = 0.03125 μg/mL). While there are no standardized, established clinical breakpoints for *Exophiala* species, the isolate reported herein was defined as resistant to echinocandins and fluconazole because the isolate was able to grow at the highest concentrations tested for these antifungals (8 μg/mL and 256 μg/mL, respectively). Similarly, the definition of susceptible was established following literature-based interpretations. If an *Exophiala* species exhibits low MICs, this can be translated to high susceptibility to the antifungal tested [1,2].

The patient refused antifungal treatment due to personal reasons. During follow-up, the nail lesions remained stable without any sign of spontaneous improvement.

## 3 Discussion

*Exophiala* species are common fungi in the environment, not only associated with decaying wood and soil enriched with organic waste, but they can also be found in warm household environments with high humidity and rich in aromatic hydrocarbons, such as steam baths, faucets, and dishwashers [2]. Therefore, the recovery of *Exophiala* species from clinical samples must be interpreted with caution, as they can be contaminants. In the case of our patient, *E. alcalophila,* a slow-growing fungal species, was definitely considered the etiological agent of the onychomycosis affecting her big toenails, based on various criteria: i) the affection of both left and right big toenails, ii) the presence of abundant and unique colonies after culturing nail scrapings from both toenails, iii) the absence of other fungi in the samples and the cultures, after 30 days of incubation, which proves that an aseptic technique was used to prevent the growth of contaminants, and iv) the fact that *E. alcalophila* is among the species of the genus that is very seldom recovered from nature and household environments, which suggests a true infection rather than contamination [2,6].

Being an infrequent human pathogen brings other connotations, mainly regarding the diagnosis and identification. As in our case, it is possible not to observe *Exophiala* during a direct nail examination, not only because very few fungal cells could be present in the nail, but also because these fungi are polymorphic, making them sometimes difficult to distinguish from standard cellular debris [17]. KOH preparation is expertise-dependent, and an adequate amount of specimen is critical in ensuring its success, with sensitivity values reported as low as 48 to 60% [18]. Unlike onychomycosis by dermatophytes, which form extensive networks of hyphae, *Exophiala* infections tend to have very sparse fungal elements scattered throughout the tissue, which decreases the sensitivity of direct microscopic examination [6,17]. The use of morphological and biochemical characteristics of *Exophiala* species after culture, which can take up to several weeks, is also not the best approach for species identification, considering that species can be erroneously described [1]. In addition, commercially available technologies such as MALDI-TOF mass spectrometry can fail to identify *Exophiala* species, mainly because of their complex cell walls, which make additional sample preparation steps necessary, or, as in the case of our isolate, because of the limited commercial databases that do not include dematiaceous yeast species. In this way, since the genus *Exophiala* exhibits diverse phenotypic traits and close genetic relationships, molecular diagnostics remains the most definitive method, thus, species identification is definitely achieved by ITS sequencing [6]. Unfortunately, due to higher costs and the need for dedicated personnel, ITS sequencing is generally limited to academic, reference, or specialized mycology laboratories rather than routine dermatology laboratories and clinics.

Onychomycosis due to dematiaceous fungi of the genus *Exophiala* is exceptional. After a comprehensive and systematic review, only 12 cases were found to be reported in the literature. Four cases by *E. dermatitidis*, three cases each by *E. jeanselmei* and *E. oligosperma*, and one case each by *E. bergeri* and *E. hongkongensis*, without the report of *E. alcalophila* [3,[7], [8], [9], [10], [11], [12],^14^] (Table 1). In the case of our patient, previous onychocryptosis is likely to have predisposed to fungal infection, considering that not only the ingrown nails *per se*, but the recurrent physical procedures on the affected nails, pierce the surrounding skin and nails, creating trauma, dystrophic nails changes and wounds that lead to an inflammatory reaction and tissue damage, making the area highly susceptible to opportunistic fungi to easily invade the nail bed [17]. When onychomycosis is encountered in big toenails, especially the bilateral presentation as in the case of our patient, infections typically require systemic treatment rather than topicals alone to achieve a complete cure, because these toenails are thick and grow slowly [5,17]. Furthermore, the treatment of non-dermatophyte onychomycosis is not well standardized and can be difficult, particularly due to resistant isolates and poor treatment adherence. While currently, systemic antifungals such as itraconazole are used to successfully treat nail infections by *Exophiala*, as reported in three of the revised cases from the literature [[10], [11], [12]], their use requires careful clinical monitoring. The extended treatment courses with itraconazole, which can take up to 6 months until full recovery of the nail, may cause patients not to respond well to treatment, as reported in one case [14], mainly because of the side effects of this triazole drug.

The heterogeneous and changing epidemiology of fungal infections, the emergence of intrinsically resistant saprophytic species as human pathogens, the difficulties in species identification, and the observations indicating an increasing relevance of non-dermatophyte fungi causing onychomycosis should have an impact in calling attention to a better diagnosis and choice of treatment of these superficial infections [6]. Importantly, dermatologists should be aware that onychomycosis can trigger more complex infectious lesions in other parts of the body. Further studies are needed to elucidate the natural habitat of *E. alcalophila* and other black yeast-like fungi, to determine the possible transmission and infection routes, and to estimate the real prevalence of infections caused by emerging fungal pathogens, which could be highly underestimated.

Lastly, while the differential diagnosis for onychomycosis spans inflammatory, malignant, genetic, and traumatic conditions that present with discoloration, thickening, or nail separation, it is important to consider that *Exophiala* species are well-documented pathogens and clinical mycology cases have reported onychomycosis where *Exophiala* is the distinct, causative agent of nail damage [3,[7], [8], [9], [10], [11], [12],^14^]. In addition, although for this study the diagnosis of onychomycosis relied only on fungal culture, this was considered sufficient, as it is consistent with daily practice, where single confirmatory tests are performed, particularly when using fungal cultures, which have a specificity of 83 to 100% [17,18]. Repeated isolation of *E. alcalophila* from independent specimens would provide stronger evidence supporting its etiological role in this case, however, we consider that we avoided false-positive results by taking an adequate amount of scrapings and following aseptic techniques to prevent contamination. The absence of dermatophytes, yeasts, and other common environmental fungi in the culture, which instead yielded an abundant, pure growth of black yeast-like fungi, proves as well the true clinical significance of *E. alcalophila* infection.

## CRediT authorship contribution statement

**Lili Rueda-Jaime:** Investigation, Resources, Writing – original draft, Writing – review & editing. **Leticia Sopó-Prada:** Conceptualization, Investigation, Methodology, Writing – review & editing. **Carolina Firacative:** Data curation, Formal analysis, Funding acquisition, Methodology, Resources, Software, Writing – original draft.

## Ethical Form

Submitted via the Ethical Form.

## Conflict of interest

There are none.
